# Placental Nutrient Transport and Intrauterine Growth Restriction

**DOI:** 10.3389/fphys.2016.00040

**Published:** 2016-02-16

**Authors:** Francesca Gaccioli, Susanne Lager

**Affiliations:** Department of Obstetrics and Gynaecology, University of CambridgeCambridge, UK

**Keywords:** fetal growth, nutrient allocation, amino acids, glucose, lipids, placental insufficiency

## Abstract

Intrauterine growth restriction refers to the inability of the fetus to reach its genetically determined potential size. Fetal growth restriction affects approximately 5–15% of all pregnancies in the United States and Europe. In developing countries the occurrence varies widely between 10 and 55%, impacting about 30 million newborns per year. Besides having high perinatal mortality rates these infants are at greater risk for severe adverse outcomes, such as hypoxic ischemic encephalopathy and cerebral palsy. Moreover, reduced fetal growth has lifelong health consequences, including higher risks of developing metabolic and cardiovascular diseases in adulthood. Numerous reports indicate placental insufficiency as one of the underlying causes leading to altered fetal growth and impaired placental capacity of delivering nutrients to the fetus has been shown to contribute to the etiology of intrauterine growth restriction. Indeed, reduced expression and/or activity of placental nutrient transporters have been demonstrated in several conditions associated with an increased risk of delivering a small or growth restricted infant. This review focuses on human pregnancies and summarizes the changes in placental amino acid, fatty acid, and glucose transport reported in conditions associated with intrauterine growth restriction, such as maternal undernutrition, pre-eclampsia, young maternal age, high altitude and infection.

## Introduction

Intrauterine growth restriction (IUGR) is defined as the failure of a fetus to reach its genetically determined growth potential (Brodsky and Christou, 2004). IUGR affects approximately 5–15% of all pregnancies in the United States and Europe, but varies widely among developing countries (30–55% of infants born in South Central Asia, 15–25% in Africa, and 10–20% in Latin America; Kramer, 2003; Saleem et al., 2011). Identification of fetal growth restricted infants is made difficult by the lack of international consensus on the definition and diagnostic criteria for IUGR. In clinical practice detection of IUGR fetuses is based on weight at birth (<2500 g) or estimated fetal weight (<10th percentile), and by ultrasound assessments of fetal growth (abdominal circumference <2.5th percentile). Moreover, altered Doppler velocimetry indices, such as abnormal umbilical artery waveforms or decreased pulsatility of the middle cerebral artery, suggest abnormalities in the fetal circulation and are indicative of IUGR. Notably, the majority of small-for-gestational age (SGA) babies are constitutionally small and healthy, while only 10–15% of SGA infants are growth restricted, i.e., with slow growth velocity in utero (Alberry and Soothill, 2007). Likewise, fetuses with falling growth trajectories might be IUGR cases, yet not SGA (Alberry and Soothill, 2007). However, it is not always possible to establish whether an observed low birth weight results from in utero growth restriction and evidence of low birth weight has been often used as a proxy for IUGR. In fact, SGA infants with birth weight below the 2nd centile for gestational age are at higher risk of being growth restricted (de Jong et al., 1998).

IUGR is associated with an increased risk of stillbirth (Smith and Fretts, 2007), while also a major cause of perinatal morbidity and mortality (Salam et al., 2014). IUGR infants are at higher risk of preterm birth, asphyxia, altered thermoregulation, hypoglycemia, cardiac dysfunction, and infections. Moreover, reduced fetal growth may have adverse consequences on lifelong health, including impaired neuro-developmental progress in childhood and higher risk for metabolic and cardiovascular diseases in adulthood (Barker, 2006; Longo et al., 2013).

## Placental nutrient transport

**Figure 1:**
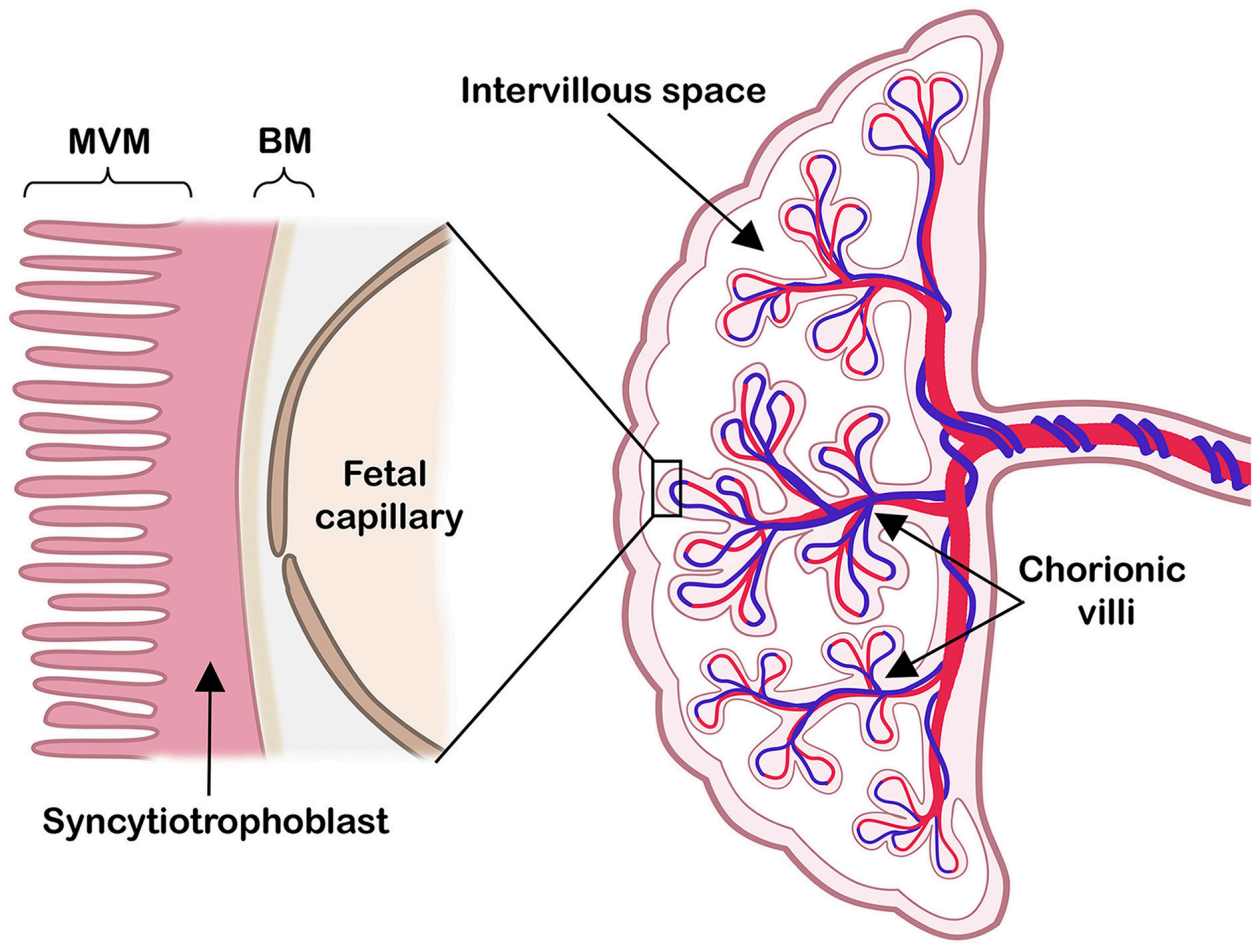
**The human placenta at term**. Right: a schematic cross-section of the human term placenta with the fetal circulation in umbilical cord and chorionic villi, while maternal blood pools in the intervillous space. Left: representation of the placental barrier, which includes the syncytiotrophoblast layer and the fetal capillary endothelial cells. The two polarized syncytiotrophoblast plasma membranes, the microvillous plasma membrane (MVM) and the basal plasma membrane (BM) are indicated. Modified illustration from Nordisk Nutrition (Lager, 2013), re-printed with permission.

As the interface between mother and fetus, the placenta mediates exchange of nutrients, oxygen and waste products, thereby ensuring proper fetal growth and development. In term human placenta essentially two cell layers (fetal capillary endothelium and syncytiotrophoblast) separate fetal and maternal circulations. The syncytiotrophoblast has two polarized plasma membranes: a microvillous membrane (MVM) directed toward the intervillous space and a basal membrane (BM) facing the fetal capillaries (Figure 1).

Various factors influence transfer of substances between maternal and fetal circulations. These factors include utero-placental and umbilical blood flow, concentration gradients of nutrients, as well as the thickness, exchange area, and metabolism of the placenta. The transfer of membrane permeable molecules, such as oxygen and carbon dioxide, is greatly influenced by blood flow and placental structure (Gude et al., 2004). Larger and less membrane permeable substances rely on transporter-proteins to cross the placenta, in a process fueled by ionic gradients (Lager and Powell, 2012).

Both syncytiotrophoblast plasma membranes express several *amino acid transporters* (Cleal and Lewis, 2008). Amino acid transporters can be categorized according to substrate specificity (system), sequence homology (family), or physiological function (accumulative transporters or exchangers). Accumulative transporters increase intracellular amino acid concentrations by mediating uptake of specific amino acids into the syncytiotrophoblast, usually by co-transporting Na^+^ (e.g., System A, transporting non-essential amino acids such as glycine and alanine, and System β, transporting taurine). Exchangers alter the intracellular composition without modifying the total amount of amino acids. In general, exchangers substitute non-essential amino acids for essential amino acids (e.g., System L; Broer, 2002). In human placenta, about 20 different amino acid transporters have been identified. Both accumulative and exchanger transporters are expressed in the MVM. Efflux of amino acids from the syncytiotrophoblast toward the fetal circulation is less well characterized. It has been suggested the BM expresses transporters allowing for facilitated diffusion, in addition to the accumulative and exchange transporters (Cleal and Lewis, 2008).

*Fatty acids* cross the placenta in a multistep process. Lipases associated with the MVM hydrolyze triglycerides into non-esterified fatty acids; the placenta expresses several lipases, including lipoprotein lipase (Herrera and Ortega-Senovilla, 2014). Then the membrane-bound fatty acid transport proteins (FATPs) mediate uptake of long-chained fatty acids (Kazantzis and Stahl, 2011). Five different FATP isoforms are expressed in human placenta (Schaiff et al., 2005). Human placenta also expresses CD36 (fatty acid translocase) (Campbell et al., 1998), a receptor proposed to transport fatty acids or sequester them close to cell membranes in order to facilitate their uptake by FATPs (Schwenk et al., 2008). CD36 and FATPs are expressed in both MVM and BM (Campbell et al., 1998; Dube et al., 2012), suggesting an involvement in syncytiotrophoblast fatty acid uptake and efflux. Further, fatty acids are delivered to different intracellular compartments by fatty acid binding proteins (FABPs) (Smathers and Petersen, 2011). The human placenta expresses four FABP isoforms (Biron-Shental et al., 2007). In addition, a membrane-associated version of FABP, with exclusive MVM localization, has been described (Campbell et al., 1998).

Placental *glucose transport* occurs by facilitated diffusion through specific glucose transporter proteins (GLUTs), expressed in both plasma membranes of the syncytiotrophoblast (Baumann et al., 2002). Glucose levels are higher in maternal circulation than in fetal circulation (Taricco et al., 2009), resulting in a net glucose transport to the fetus. The GLUT family contains 14 members (Mueckler and Thorens, 2013), of which several are expressed in the placenta (Lager and Powell, 2012). The expression pattern of the GLUTs varies slightly during gestation, but GLUT1 is considered the primary placental transporter of glucose (Baumann et al., 2002).

## Etiology of IUGR

**Figure 2:**
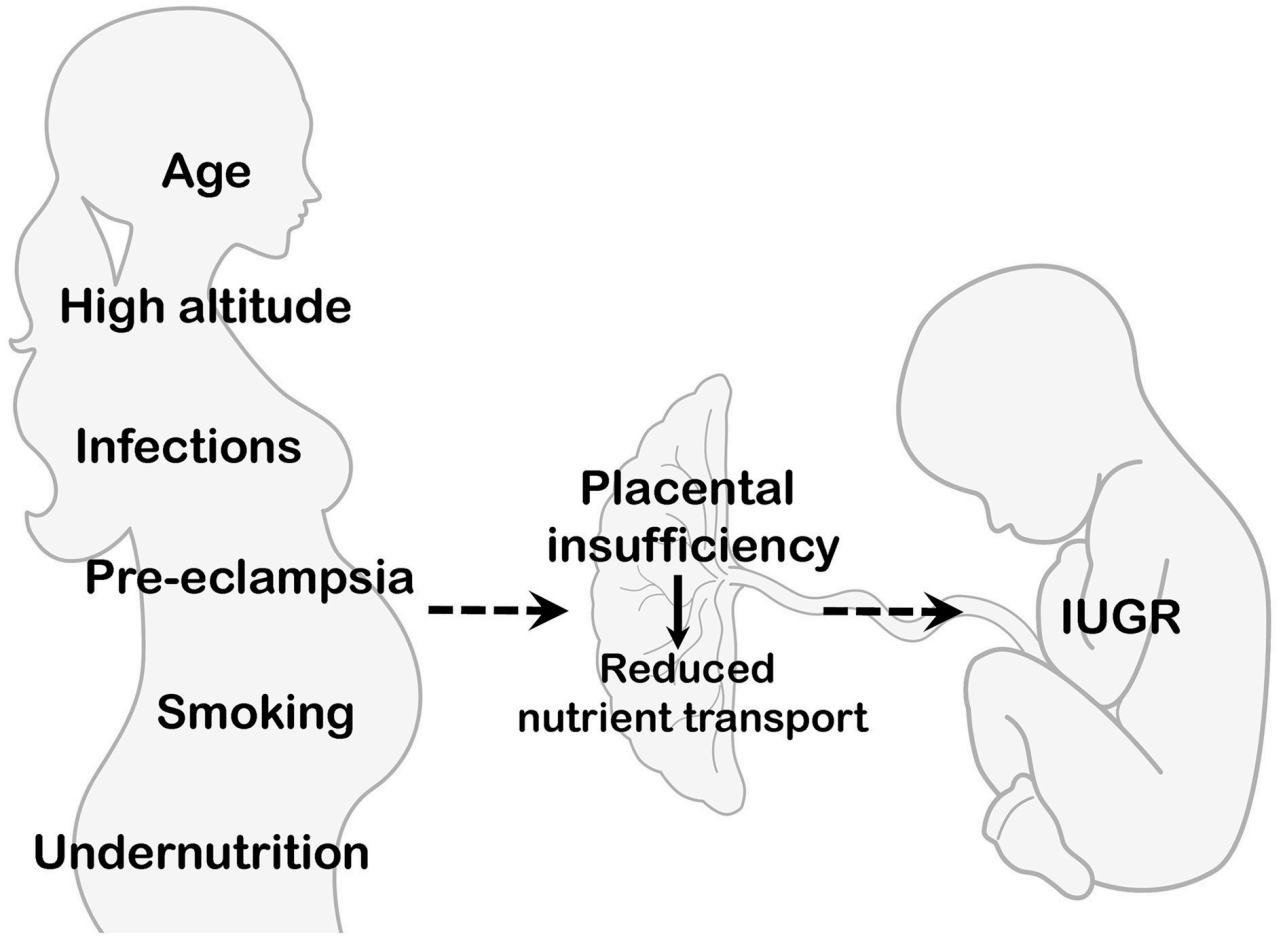
**Maternal and environmental conditions associated with IUGR**. Several maternal/environmental conditions are associated with placental insufficiency and decreased delivery of nutrients to the developing fetus. Net placental transport is determined by multiple factors, such as uteroplacental and umbilical blood flows, placental exchange area and metabolism, as well as the activity and expression of placental nutrient transporters.

IUGR is often associated with impaired placental development, structure and morphology, which in turn alter placental function and capacity of delivering nutrients to the fetus (Baschat and Hecher, 2004; Lager and Powell, 2012). Several causes, of environmental, maternal, and fetal origin, can lead to placental insufficiency. These include pregnancies at high altitude, conditions associated with altered uteroplacental blood flow, young maternal age, undernutrition, placental infections and inflammatory processes, cigarette smoke, illicit drugs use, fetal genetic diseases, and congenital malformations (Figure 2). Although optimal materno-fetal exchange of nutrients and gases is of critical importance for fetal growth, studies describing changes in placental transport capacity in the above situations are rarely available in human cohorts and we will briefly summarize them in this review.

### Maternal undernutrition

Decreased and/or imbalanced maternal nutrient availability is associated with an increased risk of delivering a SGA infant (Stein and Susser, 1975b; Ramakrishnan et al., 2012; Kozuki et al., 2015). The perinatal and long term effects of maternal undernutrition have been extensively studied taking advantage of the “natural experiment” represented by the Dutch famine, a well-defined famine period that lasted approximately 6 months (during the winter of 1944–45) in the German-occupied western provinces of the Netherlands. Studies on pregnancies during the Dutch famine demonstrated that maternal undernutrition in the third trimester inhibits placental growth (Stein and Susser, 1975a).

Maternal undernutrition due to a limited food supply is the main cause of IUGR in developing countries and constitutes a significant problem in industrialized countries where low-income households still experience “food insecurity” (Dowler and O'Connor, 2012). Other causes of low food intake and decreased availability of nutrients for the fetus are severe vomiting after the 16th week of gestation (hyperemesis gravidarum; Snell et al., 1998), short interpregnancy intervals, and adolescent pregnancies.

The impact of maternal undernutrition on fetal growth is at least in part mediated by its effect on the placenta, as this condition is associated with altered placental size, vascular development, endocrine function, and nutrient transport. Although to the best of our knowledge no data is available concerning placental nutrient transport in human pregnancies following maternal undernutrition, useful information are provided by several animal models, including nonhuman primates (Gaccioli et al., 2013). In a baboon model of global maternal nutrient restriction (MNR) during pregnancy, fetal and placental weights were reduced compared with controls. MVM expression of glucose and amino acid transporters (GLUT1, taurine transporter TAUT, SNAT2, LAT1, and LAT2) was downregulated in MNR baboons compared to controls in late gestation (Kavitha et al., 2014). Moreover, decreased transporter expression was paralleled by lower MVM activity of System A and System L in MNR baboons and reduced circulating fetal amino acid concentrations (Pantham et al., 2015).

### Pre-eclampsia

A hallmark of pre-eclampsia is new-onset of hypertension and proteinuria occurring at or after 20 weeks of gestation. IUGR is often an adverse perinatal outcome of pre-eclampsia (Chaiworapongsa et al., 2014) and the two conditions share certain traits, such as inadequate re-modeling of maternal spiral arteries (Brosens et al., 2002). However, the etiologies of IUGR and pre-eclampsia may differ (Villar et al., 2006).

With respect to nutrient levels, alterations in maternal and fetal circulations have been reported in pre-eclamptic pregnancies. Maternal amino and fatty acid blood levels are elevated in pregnancies complicated by pre-eclampsia (Evans et al., 2003; Alvino et al., 2008). Furthermore, total amino acid concentrations are higher and inversely correlate with fetal growth in cord blood from pre-eclamptic pregnancies (Evans et al., 2003). Alterations in cord blood fatty acids levels with pre-eclampsia have also been reported, suggesting lowered omega-3 fatty acids and elevated monounsaturated fatty acid levels (Roy et al., 2014).

Several studies reported altered activity or mRNA levels of placental nutrient transporters in association with pre-eclampsia. Specifically, placentas from pre-eclamptic pregnancies have a reduced capacity to transport the essential amino acid taurine (Desforges et al., 2013). While placental mRNA levels and activity of System A transporters are not affected, arginine transport across the BM is increased in placentas from pre-eclamptic pregnancies (Speake et al., 2003; Malina et al., 2005; Shibata et al., 2008). Hence, pre-eclampsia has differential effects on several amino acid transporters found in placenta. Expressions of placental fatty acid transporters are altered by pre-eclampsia as well. Placental mRNA levels of FATP1 and FATP4 are lower in pre-eclampsia compared to healthy pregnancies, while CD36 and lipoprotein lipase levels remain unchanged (Laivuori et al., 2006; Wadhwani et al., 2014). Whether protein levels and/or activities are affected similarly remains to be established.

### Maternal age

Adolescent pregnancies are associated with an increased incidence of low birth weight and SGA infants along with other adverse outcomes and maternal/perinatal mortality (Fraser et al., 1995; Shrim et al., 2011; Weng et al., 2014). Although several socio-demographic factors may play a role in the poor outcomes of adolescent pregnancies, the biological immaturity of these mothers is an independent risk factor (Fraser et al., 1995). It has been proposed that this effect may be due to the maternal young gynecologic age and continued growth during pregnancy. This can impact upon uterine blood supply and govern competition for nutrients between the mother and fetus. However, it is still controversial whether maternal growth is associated with preferential partitioning of nutrients to the still growing mother and, consequently, poor pregnancy outcomes (Stevens-Simon and McAnarney, 1993; Scholl et al., 1994; Frisancho, 1997; Jones et al., 2010).

Work by Hayward and colleagues demonstrated that although placental growth and development were not influenced by maternal age, teenage mothers had lower placental System A activity and reduced mRNA expression of the System A isoforms SNAT1 and SNAT2 compared to adult mothers (Hayward et al., 2011, 2012). This result may partially explain the higher risk of delivering SGA infants in adolescent pregnancies, although in this small UK study cohort there were no significant differences in System A activity between placentas of appropriate-for-gestational age (AGA) and those from SGA infants delivered to teenagers. When stratifying adolescent mothers into growing and non-growing, placental System A activity was comparable in the growing teenagers and adult mothers, while non-growing teenagers had significantly lower placental System A activity compared to the other two groups. Such data suggests that maternal growth is not detrimental in nutrient partitioning to the fetus, although it is not completely clear whether the non-growing adolescents in these studies were skeletally mature or poorly nourished. In the latter scenario a lower placental System A activity could indicate an attempt to spare amino acids for the undernourished mother (Hayward et al., 2011, 2012).

### High altitude

Risk of reduced birth weight is increased in pregnancies at high altitude (Jensen and Moore, 1997; Krampl et al., 2000; Giussani et al., 2001), but in populations with multigenerational high altitude residence (Andean and Tibetan) birth weight decline is less than populations with shorter residence time (Europens and Han; Moore, 2001; Moore et al., 2011). However, it is still controversial whether impaired oxygen delivery to the fetus contributes to reduced fetal growth in women of European ancestry at high altitude compared to their Andean counterparts. The team led by Moore suggested that a greater increase in uteroplacental blood flow and oxygen delivery, observed as early as 20 weeks of gestation, contributed to the higher birth weight observed in pregnancies of Andean compared to European mothers (Vargas et al., 2007; Wilson et al., 2007). Although Zamudio and co-workers confirmed reduced uterine artery blood flow in high altitude pregnancies of Europeans compared to Andeans, they proposed that maternal oxygen delivery was similar between ancestry groups due to higher maternal hemoglobin content and hematocrit in European mothers (Zamudio et al., 2007). Moreover, umbilical blood flow and absolute oxygen delivery were lower in pregnancies of European women, but their fetuses had increased venous to arterial oxygen extraction (Postigo et al., 2009). These observations led Zamudio and co-workers to exclude that decreased fetal oxygen delivery is associated with differences in fetal growth between the two populations.

Independent of ancestral origin, at high altitude placental weight and size are not altered (Postigo et al., 2009; van Patot et al., 2009). Therefore, these placentas are larger in relation to fetal size, and have increased branching and diameter of fetal capillaries compared to their sea-level counterparts (Ali et al., 1996; Espinoza et al., 2001). Such morphological changes, together with a thinner villous membrane (Jackson et al., 1985), would facilitate placental diffusion in high altitude pregnancies.

To our knowledge no studies on placental amino acid transporter in human pregnancies at high altitude have been performed. In contrast, lower BM expression of the glucose transporter GLUT1 at >3000 meters compared to 1600 meters or 400 meters might indicate that placental glucose transport capacity is downregulated in pregnancies at high altitude (Zamudio et al., 2006, 2010). Moreover, Zamudio et al. reported an increased placental glucose consumption at high altitude and suggested that placental anaerobic metabolism spares oxygen but limits glucose availability for fetal growth (Zamudio et al., 2010).

### Infection and inflammation

Fetal growth may also be impaired by placental inflammation (villitis) and infection. Villitis, described as infiltration of inflammatory cells into placental villi, has been reported to be more common in placentas of SGA or IUGR fetuses than in AGA fetuses (Derricott et al., 2013). As with placental inflammation, certain bacterial and viral infections during pregnancy have been associated with reduced fetal growth (Brocklehurst and French, 1998; Adams Waldorf and McAdams, 2013; Pereira et al., 2014). However, whether villitis, bacterial, or viral infections are associated with altered placental capacity to transport nutrients remains to be determined.

Malaria is an infectious disease caused by the parasitic protozoa *Plasmodium*. Malarial infection during pregnancy is associated with reduced birth weights (Umbers et al., 2011; Griffin et al., 2012). Moreover, malarial infection affects uterine and umbilical artery blood flows (Griffin et al., 2012) and impairs placental capacity of transporting nutrients to the fetus. Specifically, System A activity is reduced in the placental MVM membrane and glucose transporter GLUT1 expression is lower in the BM in these pregnancies (Boeuf et al., 2013; Chandrasiri et al., 2014). Therefore, changes in blood flows and reduced nutrient transport across the syncytiotrophoblast may contribute to lower birth weights associated with placental malaria infection.

### Alcohol, smoking, and cocaine

Many women consume alcohol during pregnancy (O'Keeffe et al., 2015). While birth weights may not be affected by a low-moderate alcohol exposure, impaired perinatal growth is a characteristic of the fetal alcohol syndrome (O'Leary, 2004; Henderson et al., 2007). In diverse placental *in vitro* models, ethanol exposure has been shown to reduce taurine (System β) transport (Lui et al., 2014) and transfer of the fatty acids α-linolenic and DHA (Haggarty et al., 2002). Contrasting, ethanol does not affect placental glucose or System A amino acid transport (Schenker et al., 1989a).

About 10% of women in Europe and United States smoke during pregnancy (Baba et al., 2013; Dhalwani et al., 2014; Yang et al., 2014). Maternal smoking is associated with reduced birth weights (Andres and Day, 2000; Baba et al., 2013; Iñiguez et al., 2013; Wang et al., 2014). Similarly, the use of snuff tobacco is also associated with an increased risk of delivering a SGA infant (Baba et al., 2013). Maternal tobacco usage has been shown to have several effects on the placenta, such as reduced mitochondrial function (Bouhours-Nouet et al., 2005), increased double-stranded DNA breaks (Slatter et al., 2014), and lower volume of placental capillaries (Burton et al., 1989). With regards to placental amino acid transport, nicotine reduces the transport of arginine (Pastrakuljic et al., 2000) and inhibits System A amino acid transport (Fisher et al., 1984), but conflicting findings have been reported (Schenker et al., 1989b). Transport of fatty acids appears to not be affected by maternal smoking (Haggarty et al., 2002).

Maternal use of cocaine is also associated with reduced birth weights (Bateman and Chiriboga, 2000; Keegan et al., 2010). In perfused placental cotyledons, cocaine reduces transport of arginine, phenylalanine, and valine (Pastrakuljic et al., 2000), but does not affect transport of alanine and lysine (Krishna et al., 1995). Whether cocaine also affects fatty acid and glucose transporters is currently unknown.

## Concluding remarks

Placental transport capacity is one of the pivotal factors affecting the net transfer of nutrients to the developing fetus. Altered placental transport of amino acids, fatty acids, or glucose has been associated with several conditions known to increase the risk of delivering a small or growth restricted infant. With the aim of ameliorating the perinatal and long term outcomes in these infants, further work is needed to understand the mechanisms regulating placental nutrient transport capacity in human pregnancies with IUGR fetuses.

## Author contributions

All authors listed, have made substantial, direct and intellectual contribution to the work, and approved it for publication.

### Conflict of interest statement

The authors declare that the research was conducted in the absence of any commercial or financial relationships that could be construed as a potential conflict of interest.
